# Application time for postoperative wound dressing following breast augmentation with implants: study protocol for a randomized controlled trial

**DOI:** 10.1186/s13063-014-0529-5

**Published:** 2015-01-27

**Authors:** Denise de Almeida Mendes, Daniela Francescato Veiga, Joel Veiga-Filho, Fernando Elias Martins Fonseca, Luiz Francisley de Paiva, Neil Ferreira Novo, Ana Beatriz Alkmin Teixeira Loyola, Lydia Masako Ferreira

**Affiliations:** Division of Plastic Surgery, Universidade Federal de São Paulo, Rua Napoleão de Barros, 715,-4° andar, São Paulo - SP, CEP 04024-002 Brazil; Division of Plastic Surgery, Department of Surgery, Universidade do Vale do Sapucaí, Avenida Alfredo Custódio de Paula, 320, Pouso Alegre - MG, CEP 37550-000 Brazil; Department of Microbiology, Universidade do Vale do Sapucaí, Avenida Alfredo Custódio de Paula, 320, Pouso Alegre - MG, CEP 37550-000 Brazil; Department of Bioestatistics, Universidade do Vale do Sapucaí, Avenida Alfredo Custódio de Paula, 320, Pouso Alegre - MG, CEP 37550-000 Brazil

**Keywords:** Breast, Prosthesis implantation, Augmentation mammaplasty, Postoperative care, Dressings, Surgical wound infection, Bacterial growth, Patient satisfaction

## Abstract

**Background:**

Breast augmentation with silicone implants is one of the most frequently performed cosmetic surgeries worldwide. Surgical site infection (SSI) remains an important complication of this procedure. One of the most important risk factors for SSI is the presence of microorganisms on the skin surrounding the wound. Guidelines by the Centers for Disease Control (CDC) recommend that surgical wounds be covered with a sterile dressing for 24 to 48 hours. However, a recent study showed that the application of a dressing for six days after breast reduction reduced wound colonization by coagulase-negative staphylococci.

**Methods/Design:**

A randomized clinical trial was designed to assess two protocols of postoperative wound care to determine how the application duration of the postoperative dressing influences wound colonization in patients undergoing breast augmentation with silicone implants. Women aged between 18 and 60 years who are candidates for breast augmentation with silicone implants will be randomly allocated to group I (n = 48), in which the dressing will be removed on the first postoperative day, or group II (n = 48), in which the dressing will be removed on the sixth postoperative day. Cutaneous colonization will be assessed by cultures of samples of skin flora taken from the wound region. The incidence of SSI, using standardized CDC criteria, and the perceptions of patients towards the dressing will be secondary outcomes.

**Discussion:**

An important component of SSI prevention is to minimize all possible risk factors, and the application of postoperative dressing plays a key role in this endeavor. The results of this clinical trial may help to standardize postoperative wound care after breast augmentation with silicone implants.

**Trial registration:**

This trial was registered on 12 March 2012 with ClinicalTrials.gov (identifier: NCT01553604).

## Background

The popularity of breast augmentation with silicone implants has grown exponentially in the last decade. Since 2007, this operation is one of the most frequently performed cosmetic surgeries worldwide. In 2012, over 330,000 new breast augmentations were performed in the United States alone [1]. Despite advances in the surgical technique of breast augmentation and the technology for manufacturing breast implants, surgical site infection (SSI) remains an important complication of this procedure [2,3]. SSI increases morbidity, financial and personal costs, and the possibility of operative failure, as the implant may need to be removed to treat the infection [4].

The risk of postoperative SSI in primary augmentation mammoplasty with silicone implants is estimated to be between 0.1 and 2.5% [2,4-10]. These low rates of SSI make correlations with risk factors or specific interventions difficult, as very large sample sizes are required to determine statistically significant correlations [11]. However, due to the severity of the consequences of implant-related SSI, any contribution towards understanding these risk factors will aid in the development of strategies for preventing infection and improving surgical outcomes [12].

One of the most important risk factors for SSI is the presence of microorganisms on the skin surrounding the wound [4,11,13,14]. This risk increases when alloplastic material is used [4]. The ability of endogenous skin bacteria to colonize mammary prostheses has been demonstrated *in vitro* [15]. Bacteria from the endogenous flora or external sources of contamination may adhere to the implant and form an extracellular polysaccharide-rich matrix or biofilm, which surrounds the implant [16]. Bacteria aggregate and adhere to this extracellular matrix, enabling them to evade the host immune response and resist systemic antibiotics. Biofilm formation facilitates plasmid transfer among the bacteria, leading to highly virulent organisms. The minimum inhibitory concentration of antibiotics is 1,000 times greater for biofilms than for planktonic bacteria [17]. The seeding of a foreign body and the subsequent formation of a biofilm are not only difficult to diagnose, but may be very difficult to treat, especially with routine antibiotics [18]. Studies have suggested that chronic subclinical infection related to biofilms on implants may be associated with symptomatic capsular contracture, which is the leading cause of reoperation in mammary prosthetic surgery [19-24].

SSIs are related to preoperative factors (such as patient selection), intraoperative factors (such as type of surgery), and postoperative factors. Surgical wound management during the postoperative period greatly influences the risk of SSI because the skin incision serves as a gateway for microorganisms [4,20,25,26]. Bacteria can access the operative site via the incision. They may multiply and invade tissues to cause infection, depending on their number and pathogenic interaction with the host immune system [19]. However, few studies have justified the normalization of this period [26].

Care of surgical incisions has long been practiced. The use of dressings has been documented for over 4,000 years, but Joseph Lister introduced the concept of antisepsis only in the nineteenth century. The ideal dressing should protect the wound from trauma and contamination, absorb secretions, and provide the necessary compression to minimize edema and obliterate any dead space. Furthermore, the dressing covers unsightly operative wounds, thereby improving the psychological wellbeing of the patient [26,27].

The standardization of postoperative wound dressings, including those used after breast surgery, has been based on scarce and empirical evidence [28,29]. Different materials and techniques for the application of new dressings have been developed, however, conventional gauze-based dressings are still used very often [30]. A systematic review failed to prove the superiority of any one type of dressing over another type, or no dressing, and suggested a need for well-designed clinical trials to determine the ideal application time for postoperative wound dressings, an issue that remains controversial [31].

Guidelines by the Centers for Disease Control (CDC) recommend that surgical wounds be covered with a sterile dressing for 24 to 48 hours [25]. Subsequent postoperative care should be individualized; there is no ideal home-care protocol. At present, the care of postoperative wounds is not standardized and is based on the individual experience of each surgeon [25,26]. A recent study by Veiga-Filho *et al*. [32] showed that the application of a dressing for six days after breast reduction reduced wound colonization by coagulase-negative staphylococci. These authors also demonstrated that patients preferred to retain the same dressing for six days and considered this method to be safe [32].

## Methods/Design

### Study aims

A randomized clinical trial was designed to assess two protocols of postoperative wound care to determine how the application duration of the postoperative dressing influences wound colonization in patients undergoing breast augmentation with silicone implants. The secondary aims are to determine the SSI rates and the perceptions of the patients regarding the comfort, convenience and safety of the postoperative dressing. The trial will be conducted in a double-blinded manner in a university hospital in Brazil (Hospital das Clínicas Samuel Libânio, Universidade do Vale do Sapucaí). This trial has been registered at Clinicaltrials.gov (identifier: NCT01553604).

### Ethical issues

The Ethical Committee of the Universidade do Vale do Sapucaí have reviewed and approved the study protocol (reference number: 113.612). Only those patients who agree to sign informed consent forms will be included in the study.

### Inclusion and exclusion criteria

Patients will be recruited from the private practices of the first three authors (DAM, DFV and JVF). Women aged between 18 and 60 years who are candidates for breast augmentation with silicone implants via the inframammary fold approach will be eligible for the study. Patients with a body mass index (BMI) above 30 kg/m^2^, those on immunosuppressive therapy, those with diabetes, those who smoke, and those with a history of breast cancer or breast surgery will be excluded. Patients with an indication for an areolar or axillar approach and those who had the dressing wet will also be excluded.

### Sample size

On the basis of the literature, an increase in bacterial growth of 100 colony-forming units (CFUs) per plate will be considered clinically significant [32]. For a statistical power of 95% and an alpha test risk of 5%, the sample size has been estimated to be 48 patients per study arm.

### Group assignment, randomization and allocation concealment

Patients will be randomly allocated to group I (n = 48), in which the dressing will be removed on the first postoperative day, or group II (n = 48), in which the dressing will be removed on the sixth postoperative day. Allocation will be determined by a random, computer-generated sequence (Bioestat 5.0, Instituto Mamirauá, Belém, PA, Brazil). An opaque envelope with the patient number will be opened on the first postoperative day to reveal the allocation of the patient.

### Procedures and interventions

Patients will be instructed to bathe before surgery with a 4% chlorhexidine-based detergent (Rioquímica Indústria Farmacêutica Ltda., São José do Rio Preto, Brazil) [33]. In the operating room, antisepsis of the surgical site will be performed with a 0.5% chlorhexidine solution in alcohol (Rioquímica Indústria Farmacêutica Ltda., São José do Rio Preto, Brazil) [34]. The nipple areola complexes will remain uncovered during the operation; sterile tapes will not be used to cover them, since this is not the standardized procedure in our hospital. Patients will undergo breast augmentation under general anesthesia. All patients will receive prophylactic antibiotics (cefazolin - União Química Farmacêutica Nacional S.A., São Paulo, Brazil) [35].

The surgical approach will be via the inframammary fold. Implants (Silimed Indústria de Implantes Ltda., Rio de Janeiro, Brazil) will be placed in the subglandular, submuscular or subfascial position, depending on which is the best course of action for a given patient. The surgical site will be cleaned with sterile saline after the surgery. A sample of skin flora will be obtained for semiquantitative culture before the application of a dressing over the surgical incision.

The dressing will consist of four layers of cotton gauze, covered with micropore tape. The surgical team will not know to which group the patient belongs. On the first postoperative day, the allocation envelopes will be opened. If the patient is allocated to group I, the dressing will be removed, and a sample will be harvested for culture. The patient will be instructed to return to their normal personal hygiene routine and return on the sixth day for reassessment and collection of samples for culture. If the patient is allocated to group II, she will be instructed to not wet the bandage for six days, when it will be removed and a sample will be harvested for culture. Thus, samples of the skin flora will be obtained in the operating room just before placement of the dressing and after removal of the dressing. Additional samples will be obtained on the sixth postoperative day from patients in group I.

### Method of sample collection

A sterile fenestrated paper field with a standard 5 cm per 10 cm area will be placed over the surgical wound on the right breast. A sterile swab soaked in sterile saline will be passed across the predetermined area in a standardized manner. This swab will be placed in a sterile tube containing 1.0 ml of sterile saline and immediately transferred to the laboratory.

Culture methodsStandard culture methods will be used for the identification of microorganisms [36]. Aliquots of 0.2 ml from each sample will be plated on agar medium hypertonic mannitol (HiMedia Laboratories Pvt. Ltd., Mumbai, India) to culture *Staphylococcus* sp., on blood agar (HiMedia Laboratories Pvt. Ltd., Mumbai, India) to identify hemolytic colonies, on eosin-methylene blue agar (HiMedia Laboratories Pvt. Ltd., Mumbai, India) to identify *Enterobacteriaceae*, and on Sabouraud agar supplemented with chloramphenicol (HiMedia Laboratories Pvt. Ltd., Mumbai, India) (0.05 mg/ml) to detect fungi. The plates will be incubated in an aerobic atmosphere at 37°C. After 48 hours, a microbiologist will assess the plates. The lab technicians and microbiologist will not know which group each sample belongs to.

Culture results will be expressed as CFUs per plate. When a result exceeds 300 CFUs, it will be recorded as ‘>300 CFU/plate’. Staphylococci will be identified as coagulase-negative staphylococci or *Staphylococcus aureus*, on the basis of the results of Gram staining, the presence of hemolysis, and the results of the coagulase test.

### Assessment of postoperative infections and dressing duration

Patients will be evaluated weekly for four weeks, and then re-evaluated at six months and one year after the operation. The CDC definition and classification of SSIs will be used. As the surgery involves the implantation of prostheses, an SSI is defined as an infection that occurs within one year after the surgery [37]. The patients will use a five-point scale to classify the dressing in terms of comfort, practicality and safety. This assessment will be done at the postoperative week two visit for each patient.

### Outcomes

The primary outcome will be cutaneous colonization, expressed as the number of CFUs. The incidence of SSI and the perceptions of patients towards healing will be secondary outcomes. Participation will be completed at one year after surgery, after evaluation for the presence of infection.

### Statistical analysis

The Mann–Whitney U test will be used to test the homogeneity of the groups in terms of age, BMI and duration of surgery. The Friedman test will be used to compare the number of CFUs in the samples collected before, and one and six days after the application of the dressing in group I. If there is a significant difference between these times, then the Friedman test will be supplemented with the multiple comparisons test to determine at which time points significant differences appeared. In group II, the Wilcoxon test will be used to compare the number of CFUs in samples collected before the dressing and on the sixth postoperative day. The Mann–Whitney U test will be used to compare groups I and II in terms of the number of CFUs before and six days after the application of the dressing. Tests will always be applied independently for each medium used.

The Fisher test will be used to compare groups I and II in terms of the rates of infection. The Kolmogorov-Smirnov test will be used to compare the two groups in terms of the perceptions of the patients regarding the comfort, safety and practicality of the dressings. All statistical analyses will be performed using SPSS software (version 18, SPSS Inc., Chicago, United States of America). A *P* value ≤0.05 will be considered to be statistically significant.

## Discussion

As breast augmentation with silicone implants has become a common procedure, risks factors for complications associated with this procedure must be investigated in detail to improve procedural safety. As modern protocols and guidelines should be based on the principles of evidence-based medicine, well-designed randomized clinical trials are essential to guide or adjust clinical practice [28].

Attention to the skin incision in breast augmentation is warranted, as this incision acts as a gateway for microorganisms present on the surrounding skin or in external sources of contamination. Incisions for insertion of breast implants are most commonly placed in the inframammary fold, areola or axilla [38,39]. Each of these different incisions traverse through the breast ducts at different levels, providing for different potential amounts of contamination by breast duct bacteria-colonized material [40]. However, there is not a clear relationship between incision location and SSI, and literature on this issue is controversial. Wiener, in a review of 430 patients who underwent augmentation surgery, found a significantly higher incidence of capsular contracture when a periareolar incision was used, and he hypothesized that this could be due to bacterial contamination from contact with the endogenous bacteria within the breast tissue [40]. Jacobson *et al*., in a retrospective chart review of 197 primary breast augmentation patients, observed that transaxillary incisions produced the highest incidence of contracture (6.4%), followed by periareolar (2.4%) and inframammary (0.5%), with a statistically significant difference [41]. Stutman *et al*. performed a chart review on 619 patients who underwent primary breast augmentation, and found a trend of increased rate of capsular contracture when the inframammary incision was used, but this was without statistical significance [39]. The inframammary incisions have been the most commonly used for primary augmentation mammaplasty, since it is the simplest and most straightforward approach [38-40]. For this reason, this was the incision of choice for the current trial.

The use of biomaterials and the increased pathogenicity of bacteria enable colonization of the implant even when the bacterial load is relatively low. It is exceedingly difficult to control bacterial growth after implant colonization has occurred, and this difficulty tends to increase the infection risk [4]. Subclinical implant infection is related to the development of bacterial biofilms and severe capsular contracture around the implant, which are the leading causes of reoperation in breast augmentation [19]. The need to remove the implant can cause psychological trauma to the patient [2]. For these reasons, implant subclinical infection has become a focus of substantial research [19,20].

Bacteria are responsible for most postoperative infections that occur after breast augmentation when implants are used. Gram-positive cocci are part of the skin flora, especially in the upper portion of the trunk, and they account for many implant-related SSIs [42]. Cultures of mammary tissue collected during breast surgery demonstrate that these bacteria are the most frequent endogenous bacteria in the breasts [18]. Infections by opportunistic agents, such as fungi, Gram-negative bacteria and atypical mycobacteria, have also been described [43-45]. Therefore, we have opted to culture these organisms as well.

An important component of SSI prevention is to minimize all possible risk factors. The application of postoperative dressing plays a key role in this endeavor [12]. However, it is difficult to demonstrate gains related to the use of dressings for ensuring clean wounds and lowering the infection rate [46]. For this reason, we chose the rate of skin colonization, which is related to the SSI rate [4,11,13,14], as the primary focus of this study.

The opinion of the patients regarding the proposed treatment is an important factor in the quality of care. Two different surgical dressing strategies are proposed, and determining patients’ perceptions regarding their comfort, convenience and safety is a secondary goal of this study. The results of this clinical trial may help to standardize postoperative wound care after breast augmentation with silicone implants.

## Trial status

Recruitment began in April, 2012. A total of 69 patients have been enrolled in this study and have undergone breast augmentation with silicone implants. All patients will be monitored for the presence of infection until one year after their operation.

## Abbreviations

BMI: Body mass index
CDC: Centers for Disease Control and Prevention
CFU: Colony forming units
SSI: Surgical site infection
